# Epidemic Croup

**Published:** 1829-07-01

**Authors:** 


### MAISON ROYALE DE LA LEGION D'HONNEUR.

### Epidemic Croup. Angina Pellicularis.

M. Bourgeois, surgeon to the "Maison Royale de la Legion d'Honneur," has lately read a memoir before the Royal Academy of Medicine, respecting an epidemic which reigned in the above establishment nearly twelve months, and affected fifty-seven individuals, of whom five died. Some particulars of this malady are worthy of notice.

In the beginning of 1827, the mumps were epidemic, and when this disappeared, the more serious complaint of croup shewed itself in June of the same year. Two little girls were simultaneously seized in the outset of the epidemic. The first was 14 years of age, who, after complaining of general malaise and sore throat, with difficulty of deglutition, but without any fever, was attacked with dyspnœa, the voice became hoarse, with cough and croupy inspiration. The parotid and cervical glands were swelled, as were the tonsils. Eight leeches were applied to the throat, followed by cataplasms. In the evening, the prostration was extreme, and somnolency continual. The face was pale and tumid, the eyes fixed, the pupils dilated, the extremities cold. The breathing now became extremely laborious, and an emetic was prescribed, with a blister between the shoulders; but death took place the next morning.

The second case, at the same time, presented similar preliminary symptoms, except that the fever was intense, and the mumps were hardly perceptible. There was nothing to be seen on inspecting the mouth and fauces. An emetic was exhibited, soon after which the symptoms were rapidly augmented. The paroxysms of difficult respiration were tremendous, and in the course of the night, several portions of false membrane were discharged by coughing. This expulsion was followed by some relief to the breathing. In the evening of the second day, an expectoration of membraniform debris was established, mixed with mucus and some blood. A blister to the thorax. The expectoration continued, and the patient recovered, but not without the necessity of exhibiting two or three emetics to facilitate the expulsion of the false membranes.

This second case was scarcely terminated when six other girls were seized with the same complaint. In these the tonsils were swelled, and the velum palati presented pellicular spots, extending into the pharynx. There was no fever. The pellicular spots were cauterized with the muriatic acid. Every morning these false membranes or pellicles were found renewed, and they were regularly cauterised. In some of these cases, fever rose, with tussis rauca, oppression, and somnolency; but the violence of the disease was abated in ten or twelve days, and they all recovered. We cannot follow our author through the whole of this epidemy, but the false membranes bore a conspicuous figure in all the cases. One or two more instances we shall yet notice.

A young girl, ten years of age, presented one of these pellicular spots at the orifice of the left nostril, her health being otherwise good. This spot continued for nearly a week without any change. On the eighth day a plastic inflammation occupied the whole of the fossæ nasales, and false membranes soon appeared on the uvula, the tonsils, and the velum palati. On the tenth day there was ardor urinæ, and the patient complained of a pruritus in the vagina. On examination the parts were found lined with a false membrane, thick and white, exhaling a fetid odour. The original angina extended into the pharynx; but ultimately was arrested, and recovery took place. In one other case the inflammatory exudation extended to the genital organs, and to the mucous membrane of the rectum.

One more case only shall be noticed. A young girl, ten years of age, presented a chancrous sore on the lip, with coryza, and copious secretion of fetid serum. On the third day, the malady had spread to the throat and the respiratory conduits. On the sixth day, she died. On dissection, a false membrane was found lining the left nostril—the fossæ nasales were filled with sanious and fetid detritus—the tonsils were covered with thick concretions, which extended two thirds down the œsophagus. A similar concretion, two or three lines in thickness, penetrated the glottis, larynx, and trachea, diminishing gradually as it approached the bronchia.

The author does not allude to the subject of contagion in this memoir. The application of the mineral acids appears to have been very useful, and the peculiar pellicular exudation very distinct.—*Journ. Gen. de Medecine.*

---

